# Metal-organic frameworks for food contaminant adsorption and detection

**DOI:** 10.3389/fchem.2023.1116524

**Published:** 2023-01-18

**Authors:** Xueqi Guo, Lili Wang, Linjie Wang, Qingzhen Huang, Lijuan Bu, Qiang Wang

**Affiliations:** Chinese People’s Liberation Army Center for Disease Control and Prevention, Beijing, China

**Keywords:** food contamination, adsorption material, sensor, metal-organic frameworks (MOFs), Zn-based MOFs, Cu-based MOFs, Zr-based MOFs

## Abstract

Metal-organic framework materials (MOFs) have been widely used in food contamination adsorption and detection due to their large specific surface area, specific pore structure and flexible post-modification. MOFs with specific pore size can be targeted for selective adsorption of some contaminants and can be used as pretreatment and pre-concentration steps to purify samples and enrich target analytes for food contamination detection to improve the detection efficiency. In addition, MOFs, as a new functional material, play an important role in developing new rapid detection methods that are simple, portable, inexpensive and with high sensitivity and accuracy. The aim of this paper is to summarize the latest and insightful research results on MOFs for the adsorption and detection of food contaminants. By summarizing Zn-based, Cu-based and Zr-based MOFs with low cost, easily available raw materials and convenient synthesis conditions, we describe their principles and discuss their applications in chemical and biological contaminant adsorption and sensing detection in terms of stability, adsorption capacity and sensitivity. Finally, we present the limitations and challenges of MOFs in food detection, hoping to provide some ideas for future development.

## 1 Introduction

Safety problems caused by food contamination have been paid more attention recently. According to the nature of different pollutants, food contaminants can be divided into biological, chemical, and physical contamination. Biological contamination mainly includes bacteria and bacterial toxins, fungi and fungal toxins, viruses, other microorganisms, and parasites and insects caused by pollution. Chemical contamination mainly includes pesticide and veterinary drug residues, toxic metals, and organisms (e.g., lead, arsenic, mercury, chromium, phenol), food additives (e.g., nitrite, potassium sorbate), prohibited additives (e.g., sodium formaldehyde sulfoxylate, melamine, plasticizers), harmful substances produced during food processing and storage (e.g., nitrosamines, acrylamide). Physical contamination mainly includes foreign substances and radioactive material contamination. These food risk factors in raw materials, processing, transportation, preparation, storage, and supply may produce harm at any time (Hitabatuma et al., 2022). In recent years, contamination incidents, including dioxin-contaminated eggs, milk powder contaminated with melamine, and food products carrying *Salmonella*, have been reported (Wang J et al., 2019). Therefore, detection and analysis of food contamination are critical in identifying and controlling risks to ensure food safety. Many methods have been developed to detect the presence of safety hazards in food using nanotechnology (Javier et al., 2016). Most of the methods used to assess food safety, such as high-performance liquid chromatography (HPLC), inductively coupled plasma mass spectrometry (ICP-MS), and gas chromatography-mass spectrometry (GC-MS), require great human labor, expensive equipment, long detection times, bulky equipment and the others, making them less applicable in rapid detection (Chinnapaiyan et al., 2022; Zahra et al., 2022). To overcome the limitations of conventional methods, there is an urgent need to develop new rapid detection methods that are easy to use, portable, less expensive, with high sensitivity and accuracy, such as fluorescence sensing, electrochemical sensing, surface-enhanced Raman scattering (SERS) and the others. It is also important to develop new materials with the required properties to enable their integration into sensor systems. Among various materials research, metal nanoparticles (NPs), graphene oxide (GO), carbon nanotubes (CNT), molecularly imprinted polymers (MIP), quantum dots (QDs) have all been extensively investigated. These materials are used in sensor construction as components in devices tailored to detect chemical or biological contaminants (Yang L et al., 2022). Metal-organic framework materials (MOFs) are increasingly being investigated as emerging functional materials in food detection (Hashemi et al., 2017). In addition, sample preconditioning is becoming increasingly important because the matrix of animal-derived food samples is complex and often requires purification and enrichment of residual compounds in sample before testing. Currently, the commonly used methods for food sample pretreatment are organic solvent dilution and extraction, which, however, could be time-consuming and could reduce the sensitivity of the method due to the effect of organic solvents on bioactive materials (Fang et al., 2019). Therefore, it is necessary to find an efficient, rapid, and biocompatible pretreatment method. In recent years, MOFs with more adsorption properties are considered ideal adsorbents (Tokalıoğlu et al., 2017) for sample purification, and it has been increasingly used in the adsorption and purification of hazardous substances (Li Z et al., 2019; Li et al., 2020b).

**FIGURE 1:**
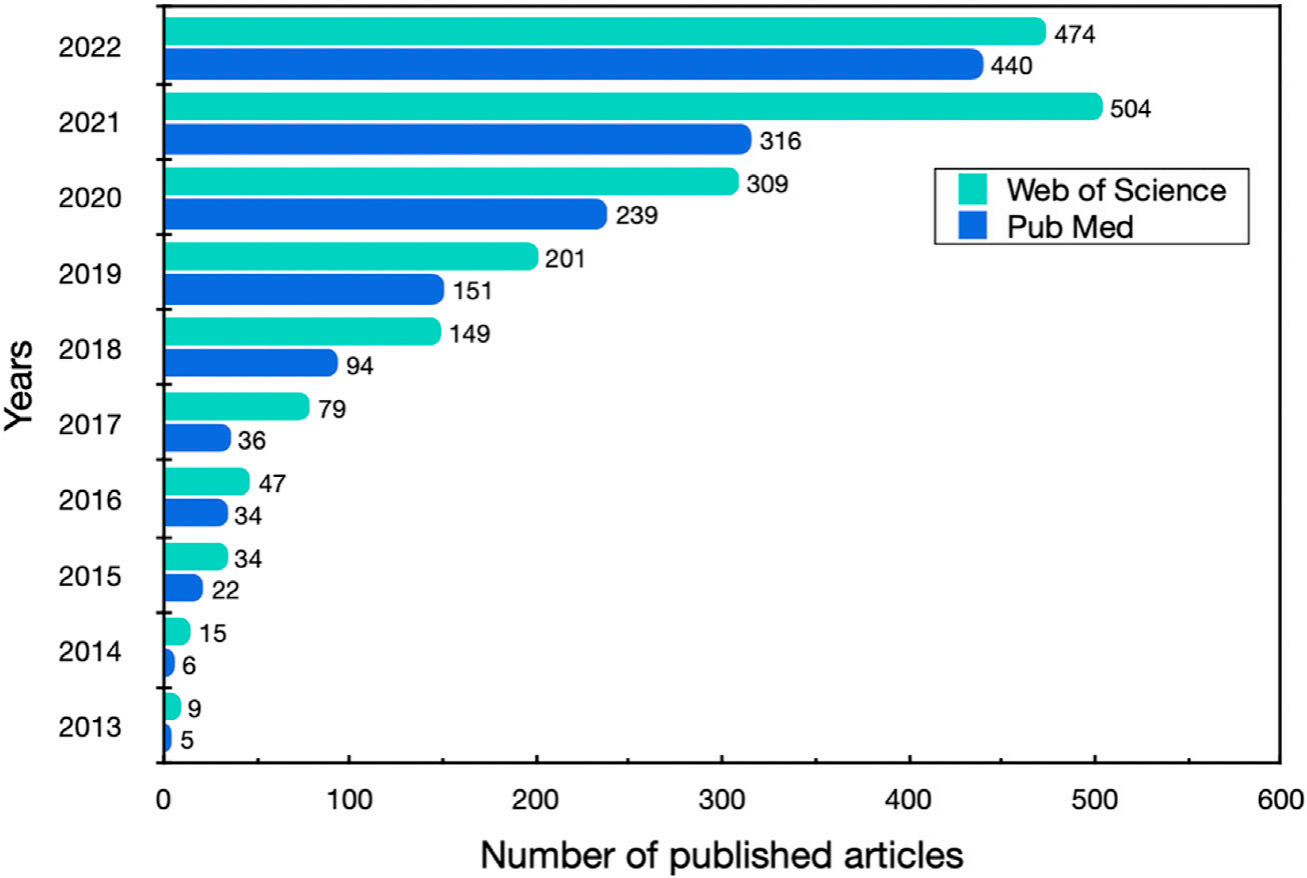
Number of published articles by MOFs in the field of food in the last decade. Data up to 17 December 2022 were obtained by searching the two databases for the keywords “metal organic framework and food.”

MOFs are a class of hybrid porous materials with infinite topological structures. At present, more than 20,000 kinds of MOFs have been reported (Jiao et al., 2019), and they have been used in fields including gas storage and release (Qin et al., 2022), catalysis (Jin et al., 2023), drug delivery (Weng et al., 2022), and contaminant removal (Ma et al., 2022a) due to their high porosity and tunable physical and chemical properties. MOFs have some special properties, such as subject-object interactions, hydrogen bonding between analyte and framework, chiral framework and analyte-specific signal response, manifesting their potential in the detection and analysis of food safety. Moreover, the application of MOFs in food is growing rapidly in recent years, with increasing number of papers being published each year, as shown in Figure 1. So far, MOFs have demonstrated their versatility, and some of them have been developed for pesticide and veterinary drug residue removal (Ma et al., 2022b), food packaging additives, food preservation, and food testing and monitoring (Li et al., 2017) for their high biocompatibility and non-reactivity with background substances (Shen et al., 2021). The unique adsorption properties of MOFs (e.g., high adsorption capacity and guest selectivity) allow them to be widely used in pre-treatment samples. The porous structure enables guest molecules to diffuse into their pores, and some special MOFs materials can be customized according to the morphology and size of the measured substance, which plays a role in concentrating the detector and eliminating impurity interference and in improving the accuracy, selectivity, and sensitivity of the detection method. Stability and recoverability are the most critical factors for the practical application of MOFs, especially in complex food matrices (Nong et al., 2020). One non-negligible drawback of MOFs is that their recoverability is lower than that of chromatographic columns (Wu et al., 2021), which can be improved by changing the ligand bonding properties, ligand conformation, and metal nodes. The stability of MOFs refers to their thermal, hydrolytic, and mechanical stability (Howarth et al., 2016). Thermal stability of MOFs is usually related to the strength of ligand bonds and number of linkers (Burtch et al., 2014). Increasing the metal-ligand binding strength using high-valent metal ions is an effective to improve thermal stability (Howarth et al., 2016). Currently, commonly used metal ions for the fabrication of chemically stable MOFs include aluminum, iron, zirconium, copper, and zinc (Jiao et al., 2019; Yuan et al., 2018). In addition, MOFs can be used as carriers to encapsulate, for example, proteins and enzymes to enhance biostability.

**FIGURE 2:**
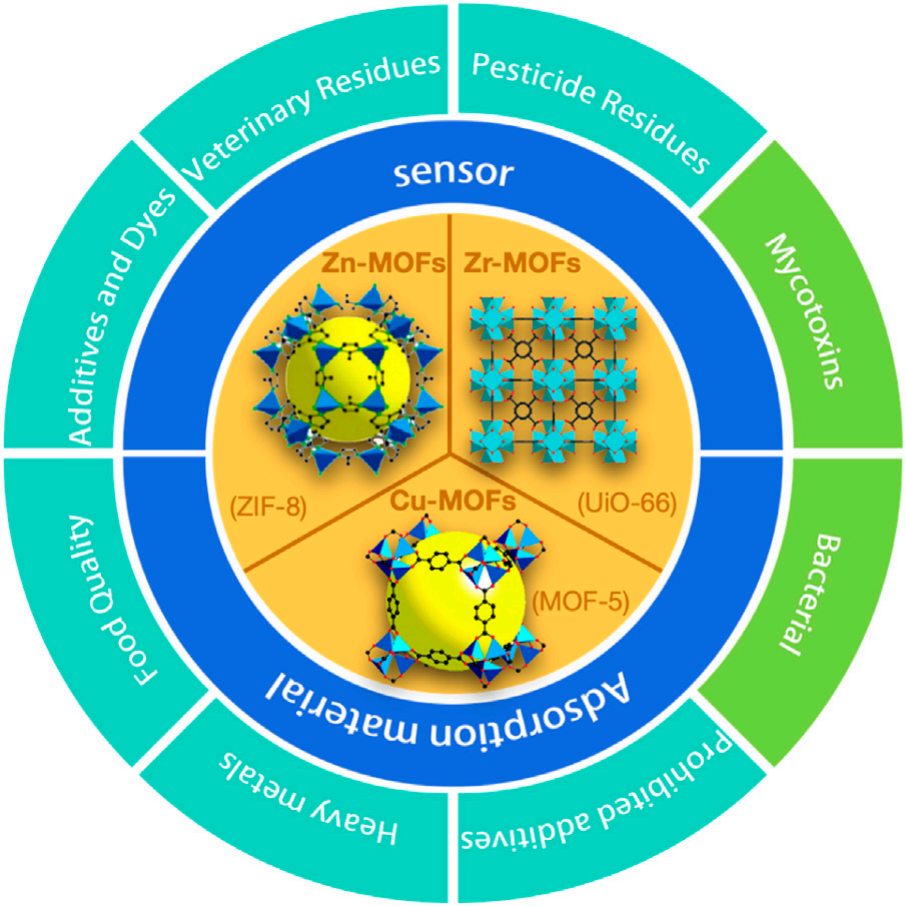
The core contention of the MOFs in this paper.

This paper reviewed the recent research progress in the adsorption and detection of food contamination from the perspective of different metal ion MOFs. Based on the preliminary literature research, the paper listed several common types of MOFs, including zinc-based, copper-based, and zirconium-based MOFs (Figure 2), which all share a common feature of easy synthesis and modification, and functionalization and are vital for their application in food detection.

## 2 Introduction of MOFs

### 2.1 Structural characteristics of MOFs

The concept of MOFs, also known as porous coordination polymers (PCPs), was first introduced by Yaghi and Li, (1995) in order to form a new crystalline material with a large surface area (typically in the range of 1,000 to 10,000 g/m^2^) by linking organic linking ligands to metal ions/clusters (secondary units SBUs). MOFs have a well-defined skeletal structure similar to that of zeolites. Unlike heterogeneous carbon materials, however, they have a very uniform pore distribution. In addition, the addition of functional groups (e.g., halogens, nitrogen, sulphur, carboxyl, cyano, nitro) to organic linkers, the selection of different metals depending on the application and the flexible adjustment of the electronic properties of the pore surface are difficult to achieve with commercially established materials such as zeolites and activated carbons (Fletcher et al., 2005). Rigid MOFs have relatively stable and robust porous frameworks with permanent porosity, similar to zeolites and other inorganic porous materials. Central metal ions/clusters play an important role in the stability of the backbone structure of MOFs. At the same time, a wide variety of organic ligands can produce MOFs with different pore sizes and pore structures (Nong et al., 2020) so that the dynamic interactions, sizes, and selectivity between pores and target guest molecules can be achieved by different organic ligands and functional groups (Deng et al., 2010). Flexible MOFs have a dynamic “soft” framework responding to external stimuli, for instance, pressure, temperature, and guest molecules, and such a sensitivity to external stimuli provides specific properties of MOFs and has been successfully combined with a variety of materials (biomolecules, carbon nanotubes, oxides, polymers, graphene, and quantum dots) to create materials with a variety of unique properties (Jiao et al., 2019).

MOF-5 [(ZnO_4_(BDC)_3_ (DMF)_8_C_6_H_5_Cl, DMF = dimethyl formamide], also named as IRMOF-1, was first synthesized by Li et al. (1999) as the most common of the IRMOFs series. MOF-5 uses terephthalic acid as an organic ligand coordination with Zn^2+^ to form a three-dimensional material in octahedral form, the specific surface area of MOF-5 is as high as 2,900 m^2^/g. The commonly studied ZIF-8 comprises transition metal ions Zn^2+^ coordinated with imidazole groups or imidazole derivatives. In the reaction, the N atom on the rigid imidazole ring loses its proton and coordinates with the transition metal ion. The combination of metal ions with imidazolyl or imidazole derivative ligands can produce different structures of ZIFs under different conditions, achieving a diverse and tunable structure. Guest@ZIFs, which are a kind of composite materials with ZIFs as the main body to load functional guest molecules, are widely studied because of their ability to enhance some properties of the main material, such as catalytic, electrochemical and fluorescent activity (Zhang X. X. et al., 2020). Although the stability of MOFs is improved by modification treatment, their pore volume and specific surface area are also reduced to some extent. Therefore, studies on the direct construction of synthetic MOFs with stability have been widely conducted. Cavka et al. (2008) synthesized UiO-66 (UiO stands for University of Oslo) consisting of Zr_6_ (*μ*_3_-O)_4_ (*μ*_3_-OH)_4_(CO_2_)_12_ metal ion cluster linked to phthalic acid. Metal ion clusters as nodes combining with organic ligands could greatly increase the porosity and specific surface area of the material, and UiO-66 also exhibits excellent stability. Due to their strong porosity and structural stability, Zr-MOFs have been intensively investigated in catalysis, molecular adsorption separation, drug delivery, fluorescence sensing, electrochemistry, and porous carriers (Bai et al., 2016).

The antimicrobial activity of MOFs may be caused by the release of metal ions or (and) through the disruption of cell membrane integrity through the active sites on material surface to degrade proteins and fatty acids on bacterial membrane. Synergistic effects (Sultana et al., 2022) can also be observed when metal ions and bioactive organic chains have antimicrobial activity. In addition, metal ions in MOFs can form coordination bond with cellulose carboxyl groups to promote the immobilization and distribution of MOFs on the surface of cellulose fibers, thereby enhancing the antimicrobial activity of biocomposites (Duan et al., 2018). Cu-based MOFs have open unsaturated Cu metal sites, and according to relevant literature, MOFs have obtained excellent antibacterial properties in the presence of Cu^2+^ (Gabriela et al., 2016). Gwon et al. (2020) conducted antibacterial tests using MOFs composed of the identical organic ligands of different metals, and the results showed that Cu-based MOFs have more potent antibacterial properties, and that the central metal of MOFs is more critical than the ligands in targeting the antibacterial effect.

### 2.2 Principle of MOFs for food contamination adsorption and detection

#### 2.2.1 MOFs as adsorption material

The ability of MOFs as adsorbents can be attributed to their great structural potential, such as central metal ions, open metal sites, linkers, porosity, and functionalization/modification of MOFs. The main advantages of MOFs are their large specific surface area, huge porosity, and easy adjustment of pore size and shape from microporous to mesoporous scale, which can lead to high extraction efficiency (Bazargan et al., 2021). MOFs clearly have better adsorption capacity and selectivity than zeolites and other materials currently commercially available (Caroline et al., 2015). The ability of MOFs to act as adsorbent materials cannot be separated from the following points: 1) A large specific surface area and pore volume that allows them to have a high adsorption capacity, while the pore size is the basis for their selective adsorption of different sized objects. 2) The active sites of MOFs can be specific for the chemisorption of certain substances. 3) Better water stability. 4) Different metal ions and diverse organic ligands make MOFs structurally diverse and therefore have the potential for selective adsorption (Yang and Gates, 2019). Analytes are often present at trace or ultra-trace levels in the sample matrix, making it difficult to obtain accurate quantitative data for target analytes using instrumental analysis alone. The porous nature of MOFs allows adsorption and pre-concentration of analytes in complex sample matrices for purification and enrichment of target analytes, therefore further improving sensing sensitivity (Xuan et al., 2022). To achieve higher selectivity, researchers usually adjust the pore size of MOFs according to the size of specific food toxin molecules. Currently, solid phase extraction (SPE), micro solid phase extraction (μSPE), magnetic solid phase extraction (MSPE), solid phase microextraction (SPME), and stir bar sorptive extraction (SBSE) techniques have been used to extract target analytes from complex sample matrices, followed by instrumental assays (Wu et al., 2021).

#### 2.2.2 MOFs as sensor

Highly active sensors composed of nanocomposites typically have high adsorption capacity, large surface area, inter-crystalline mesoporosity and low electron transfer resistance (Prathap et al., 2018). MOFs-based sensors have been widely developed. According to the different principles of action, they can be divided into fluorescent sensors, electrochemical sensors, mechanical sensors, and so on (Lei et al., 2014). Fluorescent and electrochemical sensors are currently the most studied sensors (Stassen et al., 2017; Wang X et al., 2019). Fluorescence sensors utilize the optical properties of material and convert changes into analytical signals (Marimuthu et al., 2022). MOFs-based fluorescent sensors have sensing targets, including cations, anions, small organic molecules, gases, biomarkers. In the current research, luminescent metal-organic frameworks (LMOFs) were developed according to the following approaches: 1) Luminescent molecules as ligands for MOFs for fluorescence sensing; 2) Lanthanide elements with unique fluorescence characteristics as metal ions of MOFs; 3) Doping of various functional elements into MOFs for fluorescence sensing. Some MOFs have luminescent behaviors due to their structural properties such as metal-to-ligand charge transfer (MLCT), ligand-to-metal charge transfer (LMCT), adsorption emission (Allendorf et al., 2009).

Metal ions are often used as signal tags in electrochemical assay systems because signals generated by chemical valence changes can be electrochemically detected, allowing some MOFs to exhibit excellent conductivity and high charge mobility. Many MOFs are involved in redox reactions. For efficient electrochemical sensors, two main points need to be satisfied: 1) high specificity of the signal tag and 2) high sensitivity and stability of the modified electrodes. MOFs and their composites can be designed as signal tags for electrochemical sensing because of their special structure, and water-stable MOFs are usually seen as long-term stable sensors (Yang Z et al., 2022). In addition, MOFs are ideal materials for surface modification of electrodes, and by combining MOFs with the kinetics of analytes or high-loading capacity materials, multiple properties can be integrated to improve the sensitivity of MOFs-based electrochemical sensors (Kreno et al., 2012).

## 3 Application of MOFs in food contamination adsorption and detection

### 3.1 Application of Zn-based MOFs in food contamination adsorption and detection

Zn-based MOFs usually consist of metallic ZnO or ZnO octahedral clusters as metal nodes, which can be combined with different organic ligands to form MOFs with different pore sizes. For example, the IRMOF series consists of ZnO octahedral clusters with various organodicarboxylate adapters forming different three-dimensional stereo networks with pore sizes ranging from IRMOF 1 to IRMOF 16, with a range of 12.8–28.8 Å (Bahrani et al., 2019). Another classical group of zinc-based MOFs is the ZIF series, which exhibit high thermal stability (up to 550°C in N_2_) and stability in organic solvents due to the strong bond between the central metal ion and the nitrogen atom in the ligand. ZIF-8, composed of zinc ions and 2-methylimidazole, has been extensively studied due to its unique properties such as large endopores and specific surface area (11.6 Å and 1,947 m^2^/g) (Bazargan et al., 2021).

#### 3.1.1 Zn-based MOFs as adsorption materials for food contamination

##### 3.1.1.1 Adsorption of chemical contamination by Zn-based MOFs

**FIGURE 3:**
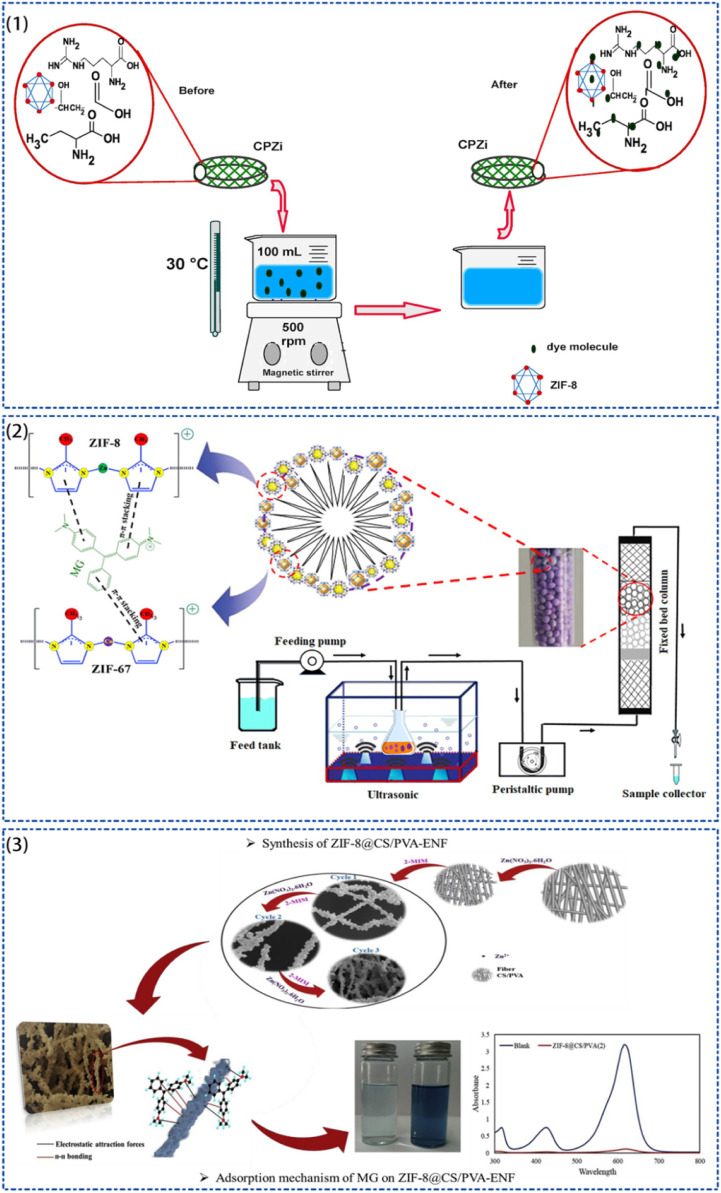
Schematic diagram of the process of MG adsorption by ZIF-8-based composites. (1) ZIF-8/CS/PVA composite membrane adsorbed MG with the maximum adsorption amount of 482.48 mg/g (Khajavian et al., 2020). (2) PES/ZIF-8/ZIF-67 microspheres adsorbed MG with the maximum adsorption amount of 613.2 mg/g (Abdi and Abedini, 2020). (3) ZIF-8@CS/PVA-ENF nanofibers adsorbed MG with the maximum adsorption amount was 1,000 mg/g (Mahmoodi et al., 2020).

Many of the analytes in food analysis pretreatments are large molecules. While most MOFs typically have relatively small pores, the small pore windows of some MOFs make it unlikely for analytes to reach the interior of the pores. However, MOFs still show the ability to extract these analytes, which can be attributed to external surface adsorption. ZIF-8 has a sodium feldspar-type Zn (mim)_2_ framework and a large cage-like (∼12.5 Å) highly hydrophobic pore system connected by small windows (Huang et al., 2006) (∼3.3 Å), which have high porosity (micropore volume: ∼0.636 cm^3^/g), large surface area (BET surface area: ∼1,630 m^2^/g), and excellent stability in organic solvents, water and highly alkaline aqueous solutions (Rahul et al., 2008). ZIF-8 shows excellent advantages in food sample detection pretreatment (Ge and Lee, 2011). Yang et al. (2013) reported the use of ZIF-8 as a column-packing sorbent for online solid phase extraction (SPE) combined with high-performance liquid chromatography (HPLC) for the detection of oxytetracycline (OTC), tetracycline (TC) and chlortetracycline (CTC) in water and milk samples. OTC, TC, and CTC are commonly found in foods of animal origin because they are broad-spectrum antibiotics used as drugs for the treatment of livestock and as additives in animal feed. Excellent analytical performance was achieved with the help of ZIF-8, with recoveries of tetracyclines in milk and water samples ranging from 70.3% to 107.4%. However, tetracyclines molecular size is larger than the ZIF-8 pore size and cannot diffuse into the pores of ZIF-8. The observed extraction performance may result from analyte adsorption on the external crystal surface of ZIF-8. ZIF-8 is strongly thermally stable, maintaining high structural stability at a temperature up to 550°C. ZIF-8 has a more abundant and direct synthesis method than other MOFs or adsorbent materials. However, it is poorly dispersed in water and tends to agglomerate, which would lead to reduced adsorption capacity (Mo et al., 2022). Therefore, ZIF-8 can be loaded on different carriers by several modifications to improve its dispersibility. Both malachite green (MG) and rhodamine B (RhB) are harmful dyes that are highly teratogenic and carcinogenic, and have side effects when absorbed by humans (Sriram et al., 2022). Abdi and Abedini, 2020 synthesized a novel polymeric nanocomposite PES/ZIF-8/ZIF-67 microspheres based on ZIFs for the removal of MG from colored wastewater, which show an excellent adsorption capacity of 613.2 mg/g of MG. Mahmoodi et al. (2020) similarly designed and synthesized a ZIF-8-based composite material and prepared ZIF8@CS/PVA-ENF nanofibers by encapsulating ZIF-8 crystals on chitosan/polyvinyl alcohol electrospun nanofibers (CS/PVA-ENF), exhibiting higher adsorption performance on MG with adsorption capacity of 1,000 mg/g and excellent chemical stability (Figure 3).

##### 3.1.1.2 Adsorption of biological contamination by Zn-based MOFs

Mycotoxins are secondary metabolites produced by fungal growth in food and feed, which are highly toxic and carcinogenic, resistant to high temperatures, chemically stable, and difficult to remove during general food processing. Han et al. (2020) synthesized a new Zn-based composite MOFs (Fe_3_O_4_@AMP&ZnCl_2_@McAbs) for multi-target adsorb deoxynivalenol (DON) (also called vomitoxin), zearalenone (ZEA), and T-2 and HT-2 toxins produced by *Fusarium* oxysporum. The results showed that the maximum adsorption amounts of Fe_3_O_4_@AMP&ZnCl_2_@McAbs per 100 mg composite were DON 688.26 ng, ZEN 864.98 ng, T-2 1,272.06 ng, HT-2 1,529.74 ng, respectively, and the recovery of the four mycotoxins decreased with the increase of the number of cycles. However, the decrease was insignificant, indicating the composites’ stability to some extent. The strong chemical and thermal stability of aflatoxin B1 (AFB1) make it more difficult to be removed from practical applications. Using reactive oxygen species with high reactivity is considered as an effective means to eliminate AFB1. Recently, Zhang X et al. (2022) developed a porous carbon material based on Fe-ZIF-8 with multiple active sites providing catalytic activity. The material can generate a large amount of highly reactive oxygen species to destroy or inactivate terminal furan ring and methoxy double bond of AFB1. Meanwhile, the large specific surface area and multi-stage porous structure of Fe-ZIF-8-PC achieve high exposure of active sites and fast mass transfer rate of reactants, enabling an excellent adsorption performance to AFB1.

#### 3.1.2 Zn-based MOFs as sensors for food contamination detection

##### 3.1.2.1 Detection of chemical contamination by Zn-based MOFs

**FIGURE 4:**
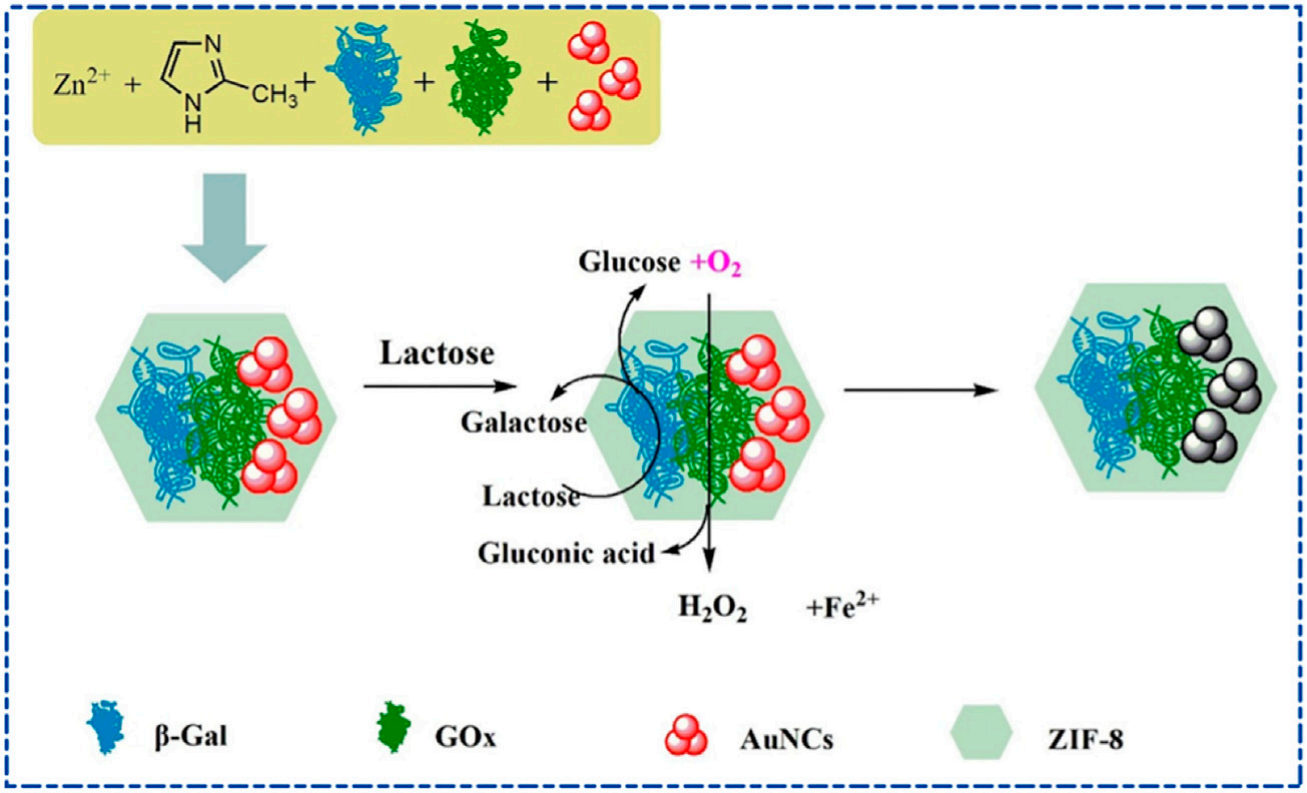
Synthesis method of AuNCs/β-Gal/GOx@ZIF-8 and measurement principle of lactose sensor (Guo et al., 2021).

Luminescent metal-organic frameworks (LMOFs) have received increasing attention in sensing (William et al., 2017). Zn-based MOFs are commonly designed as fluorescent sensors for food detection applications to detect food quality and safety (Magri et al., 2021). Pang et al. (2022) created a novel fluorescent sensor with a zinc-based porphyrin metal-organic framework (Zn-TCPP-MOF) for the ultrasensitive and quantitative detection of bisphenol A (BPA). Zn-TCPP-MOF exhibits a luminescent band in the wavelength range of 500–800 nm, probably due to ligand-to-metal charge transfer (LMCT). Meanwhile, the fluorescence spectrum of Zn-TCPP-MOF was blue-shifted compared with that of TCPP, mainly because the formation of coordination bonds between zinc ions and TCPP reduced the free electrons of porphyrin molecules, thereby increasing the lowest excited state energy level. The experiment showed that the detection limit of Zn-TCPP-MOF for BPA was 0.906 nM (0.002 mg/L), about 5 times the detection limit of GB 31604.10–2016 of the national standard for food safety in China. ZIF-8 has recently shown unique advantages in improving the suitability of enzymes. In Figure 4, Guo et al. (2021) prepared a zeolitic imidazole framework (AuNCs/β-Gal/GOx@ZIF-8) containing bovine serum albumin (BSA)-immobilized gold nanoclusters (AuNCs), *β*-galactosidase (β-Gal) and glucose oxidase (GOx) for detected lactose to improve the quenching rate of fluorescence AuNCs. Since AuNCs are only 0.8 nm in size and have BSA on their surface, BSA-immobilized AuNCs can be co-encapsulated with *β*-Gal and GOx in ZIF-8 at room temperature. When lactose is in the system, lactose is first decomposed into galactose and glucose by *β*-Gal, and glucose is decomposed into gluconic acid and hydrogen peroxide (H_2_O_2_) by GOx in the presence of O_2_ in the air. The reaction between H_2_O_2_ and Fe^2+^ generates hydroxyl radicals, leading to the fluorescence quenching of AuNCs. Based on the above principle, the detection of lactose content in lactose-free milk had a detection limit of 41.67 μM at a signal-to-noise ratio of 3, which was much lower than the threshold value of lactose-free milk samples (5 g/L, 15 mM). As the long-term storage stability of enzymes is crucial for the utility of the sensor, the authors stored AuNCs/β-Gal/GOx at ZIF-8 and the free enzyme system (AuNCs/β-Gal/GOx) for 3 days, and found that the relative activity was 76.83% and 5.30% of the initial total activity, respectively, indicating that the ZIF-8 framework material established a tremendous digestive enzyme tolerance. Meanwhile, the quenching plastic rate of AuNCs/β-Gal/GOx@ZIF-8 fluorescence by lactose was increased to 3.4 times that of the free enzyme system.

##### 3.1.2.2 Detection of biogenic contamination by Zn-based MOFs

In a previous study (Zhong et al., 2019), the authors prepared a sandwich-like electrochemical immunosensor using CdS@ZIF-8 as a novel signal probe for the ultrasensitive detection of *E. coli* O157:H7. In this system, cadmium sulfide quantum dots (CdS QDs) were encapsulated in ZIF-8 to construct a CdS@ZIF-8 complex with a core-shell structure. To specifically recognize O157:H7 cells, CdS@ZIF-8 particles introduced amino groups on the surface by wrapping them with polyethyleneimine (PEI) to enhance the interaction with antibodies. The biosensor had a linear range of 10–108 CFU/mL for O157:H7 detection and a detection limit of 3 CFU/mL (S/N = 3). The sensitivity of the proposed method was 16 times higher than that of the immunosensor using only CdS quantum dots as signal tags. The detection efficiency of the CdS@ZIF-8@PEI sensor was tested by spiking O157:H7 in milk. The results showed recoveries in the range of 94.3%–104.8%, indicating that the biosensor can be used to detect *E. coli* O157:H7 in actual samples.

Tables 1, 2 summarizes the latest research results on Zn-based MOFs for the adsorption and detection of food contaminants. With the mature development of Zn-based MOFs materials, the potential to select this material as a primary material for development is also highlighted. As ZIF-8 has a wide range of modification conditions, there is a preference for the modification and processing based on this material. From some of the studies summarized above, it can be found that Zn-based MOFs materials are excellent materials for the adsorption and detection of food contaminants due to their low LOD, high adsorption capacity, high recovery value and high detection accuracy.

**TABLE 1:** Zn-based MOFs as adsorption materials for food contamination.

Zn-based MOFs	Target analytes	Type of food contaminants	Application	Sample	LOD/AC	Ref.
**Chemical contamination**						
ZIF-8	OTC, TC, and CTC	Pesticide/Veterinary Residues	SPE	water, milk	83.6, 19.2, 12.1 mg/g	Yang et al. (2013)
Fe_3_O_4_@SiO_2_@ZIF-8/TiO_2_	triazole fungicid	Pesticide/Veterinary Residues	MSPE	water	0.19–1.20 ng/L	Su et al. (2016)
BMZIFs	organochlorine pesticides (OCPs)	Pesticide/Veterinary Residues	MSPE	tap water	0.39–0.70 ng/L	Liu et al. (2017)
ZIF-90-NPC	pyrethroid pesticides	Pesticide/Veterinary Residues	SPME	Peach, Cucumber, Cabbage	0.1–0.5 ng/g	Zhang et al. (2017)
MOF-5	Estrogens	Pesticide/Veterinary Residues	SPME	milk	0.17–0.56 ng/mL	Lan et al. (2016)
Magnetic MOF-5	PAHs and GA	Pesticide/Veterinary Residues	MSPE	food	0.91–1.96, 0.006–0.08 mg/L	Hu et al. (2013)
ZIF-8@GO	Pb II)	Heavy metals	-	water	356.0 mg/g	Wang P et al. (2019)
PHCS-15@ZIF-8	Pb(II)	Heavy metals	-	water	462.9 mg/g	Zhou et al. (2020)
Fe_3_O_4_@ZIF-8	Pb(II)	Heavy metals	-	water	719.42 mg/g	Jiang et al. (2021)
Fe_3_O_4_@ZIF-8	antioxidants	Additives and Dyes	MSPE	mineral water	0.03–0.15 ng/mL	Wang et al. (2018)
ZIF-8	putrescine (Put) and cadaverine (Cad)	Food Quality	SPME	fish	27.1 and 33.2 μg/L	Huang et al. (2017)
ZIF-8/CS/PVA	malachite green	Prohibited additives	-	water	482.48 mg/g	Khajavian et al. (2020)
PES/ZIF-8/ZIF-67	malachite green	Prohibited additives	-	water	613.2 mg/g	Abdi and Abedini (2020)
ZIF-8@CS/PVA-ENF	malachite green	Prohibited additives	-	water	1,000 mg/g	Mahmoodi et al. (2020)
**Biological contamination**						
Fe_3_O_4_@AMP&ZnCl_2_@McAbs	zearalenone (ZEN)	Mycotoxins	-	corn, wheat, and oat flour	864.98 ng/100 mg	Han et al. (2020)
Fe-ZIF-8-NPC	aflatoxin B1	Mycotoxins		wastewater from tofu	202.8 mg/g	Zhang Y et al. (2022)

LOD, Limit of Detection; AC, Adsorption capacity.

**TABLE 2:** Zn-based MOFs as sensors for food contamination detection.

Zn-based MOFs	Target analytes	Type of food contaminants	Application	Sample	LOD/AC	Ref.
**Chemical contamination**						
IRMOF-8-DPC	chloramphenicol	Pesticide/Veterinary Residues	Electrochemical sensor	honey	2.9 × 10^–9 ^mol/L	Xiao et al. (2017)
ZnPO-MOFs	parathion-methyl	Pesticide/Veterinary Residues	Fluorescent sensor	water	0.12 μg/kg (0.456 nM)	Xu et al. (2018)
[Zn_2_ (bpdc)_2_(BPyTPE)]	2,6-Dichloro-4-nitroaniline	Pesticide/Veterinary Residues	Fluorescent sensor	fruit	0.13 ppm	Tao et al. (2017)
[Zn_3_ (DDB) (DPE)]·H_2_O	2,6-Dichloro-4-nitroaniline	Pesticide/Veterinary Residues	Fluorescent sensor	carrot, grape	0.166 mg/L	Wang J et al. (2019)
ZIF-8/MB	Paraoxon	Pesticide/Veterinary Residues	Electrochemical sensor	apple and eggplant	1.7 ng/mL	Li et al. (2020a)
gCDc/AuNCs@ZIF-8	cephalexin	Pesticide/Veterinary Residues	Fluorescent sensor	milk	0.04 ng/mL	Jalili et al. (2021)
CuNCs@ZIF-8	tetracycline	Pesticide/Veterinary Residues	Fluorescent sensor	-	0.30 μM	Pirot and Omer (2022)
ZIF-8	doxycycline	Pesticide/Veterinary Residues	Fluorescent sensor	basa fish and pure milk	3.4 nM	Ding et al. (2022)
TMU-34 (-2H)	Hg(II)	Heavy metals	Fluorescent sensor	water	1.8 × 10^−6^M	Razavi et al. (2017)
AuNCs/β-Gal/GOx@ZIF-8	Lactose at lactose-free products	Food Quality	Fluorescent sensor	lactose-free milk	41.67 µM	Guo et al. (2021)
**Biological contamination**						
LMOF-241	aflatoxin B1	Mycotoxins	Fluorescent sensor	-	46ppb	Hu et al. (2015)
[Zn_2_ (tcpbp) (4.40 -bipy)_2_]	3-Nitropropionic acid	Mycotoxins	Fluorescent sensor	sugar cane	1.0 mM	Yang et al. (2020)
CdS@ZIF-8	*Escherichia coli* O 157:H7	Bacterial Contamination	Electrochemical sensor	milk	3 CFU/mL	Zhong et al. (2019)
Tb/Eu@bio-MOF-1	*Bacillus* anthracis spores	Bacterial Contamination	Fluorescent sensor	human serum	34 nM	Zhang et al. (2016)
IRMOF-3	S. arlettae	Bacterial Contamination	Fluorescent sensor	water	100 CFU/mL	Bhardwaj et al. (2016)

LOD, limit of detection; AC, adsorption capacity.

### 3.2 Application of Cu-based MOFs in food contamination adsorption and detection

Copper is a variable valence metal in transition metals, which is relatively present in large quantities and cheaper than other transition metals. Copper ions have Lewis acidity and strong electrophilic capacity. Generally, Cu-based MOFs have a large specific surface area, diverse structure, and unsaturated coordination metal centers, which greatly enhance their application in electrochemistry (Chen X et al., 2022). One of the most well-characterized and widely studied MOF materials to date is HKUST-1 (HKUST is an acronym for the Hong Kong University of Science and Technology, also known as MOF-199, CuBTC), a Cu-based MOF. It is composed of Cu ions and organic ligand benzenetricarboxylate, which has been extensively studied due to its relative ease of synthesis, excellent thermal stability and good moisture stability (Kim et al., 2012).

#### 3.2.1 Cu-based MOFs as adsorption materials for food contamination

##### 3.2.1.1 Adsorption of chemical contamination by Cu-based MOFs

Food packaging and cooking processes can introduce heavy metals into food (Wu et al., 2021), and heavy metals such as arsenic (As), mercury (Hg), lead (Pb), and cadmium (Cd) can damage human health even at trace level. Song et al. (2021) designed an adsorbent for the detection of trace amounts of total mercury in rice using the strong adsorption capacity of MOFs. Copper hydroxyphosphate (CHP) was used as a source of metal ions for the *in situ* synthesis of DMP-Cu at room temperature, and the modification of 2,5-dimercapto-1,3,4-thiadiazole (DMTZ) was found to increase the stability and effectiveness of the complex MOFs. The pH of the solution is one of the most critical factors affecting the adsorption performance. Mercury exists as Hg^2+^ when the pH is less than three and as Hg(OH)_2_ when the pH is more significant than 6.47. Both forms of mercury exist simultaneously in the pH range of 3–6.47 (Chandra and Kim, 2011). Therefore, the authors designed experiments to test the adsorption of the composite MOFs materials on mercury at pH 2–8, respectively. It has been found that the adsorption amount appeared to increase and then decrease. This was due to the presence of H^+^ and OH^−^ ions interfering with the adsorption process, and further comparison showed that the adsorption of mercury by the modified DMP-Cu increased significantly, with maximum adsorption of 249.5 mg/g at a pH value of 4. The LOD was 0.0125 ng/mL by the detection of actual samples of rice, which is well below the detection limit of Hg^2+^ concentration in the standard. In addition, the recoveries of Hg in rice samples ranged from 98.8% to 109%, proving the feasibility of the method.

##### 3.2.1.2 Adsorption of biological contaminants by Cu-based MOFs

At present, few MOFs are directly used as adsorbents for biological contaminants, possibly due to a weaker interaction between MOFs materials and biological contaminants. Carbon nanomaterials are more frequently reported due to a stronger *p*-π interactions with biogenic contaminants. It is well known that MOFs can be used as precursors to prepare porous carbon materials by simple carbonization. Some porous carbon materials derived from MOFs not only retain some excellent original properties of MOFs, but can also promote the adsorption capacity of biological contaminants. Recently, Ma et al. (2021) prepared a series of porous carbon materials (C-Cu-BTC) based on Cu-BTC for the adsorption of AFB1 from vegetable oil. The C-Cu-BTC exhibited excellent adsorption performance of AFB1 due to its enough active sites, diverse pore structure, and large specific surface area. It was found that more than 90% of AFB1 in vegetable oil could be removed within 30 min. The adsorption of AFB1 in water by the material reached 16.6667 mg/g through kinetic simulation, and the result was much higher than other reported materials.

#### 3.2.2 Cu-based MOFs as sensors for food contamination detection

##### 3.2.2.1 Detection of chemical contamination by Cu-based MOFs

Nanoscale MOFs are promising for research in electrochemical sensing. 2D MOFs exhibiting a highly ordered, pore-like arrangement at the two-dimensional scale have a larger specific surface area to facilitate the immobilization of enzymes, signal molecules, or catalysts, and can be used to achieve signal amplification (Qin et al., 2018). This signal amplification strategy was recently achieved by Ma J et al. (2022) using a combination of 2D MOFs (Cu-TCPP MOFs) and G-quadruplex and DNA nanomotors. In this report, a self-cleaning electrochemical sensor with high sensitivity and selectivity was presented for the cyclic detection of Pb^2+^ ions. This sensor detected Pb^2+^ in the linear range between 5 nM and 5 μM with a detection limit of 1.7 nM, and it can selectively detect Pb^2+^ in the presence of other metal ions. Electrochemical sensors are divided into label-free and labeled technologies. The labeled technologies use enzymes, metal nanoparticles, small molecule oxidation reducers, and metal ions as signal tags to greatly amplify the electrochemical signal. However, there are some drawbacks, for instance, the need for complex pre-enrichment steps (Zhang et al., 2009) and the vulnerability to the activity and stability of the signal tag (Liu et al., 2016). To this end, Zhang X X et al. (2020) developed a signal label-free electrochemical sensor based on Cu-MOFs material for determining Hg^2+^ in dairy products. The sensing assay was based on the specific recognition between Hg^2+^ and T-rich DNA strands to form a stable T-Hg^2+^-T coordination chemistry, which effectively eliminates the interference of the sample matrix and substantially improves the accuracy of the assay results. The presence of a large amount of Cu^2+^ in the probe eliminated the need for a complex metal pre-enrichment process. During the experiment, it was found that there was no significant effect on the Hg^2+^ signal response when interfering ions were introduced.

##### 3.2.2.2 Detection of biogenic contamination by Cu-based MOFs

**FIGURE 5:**
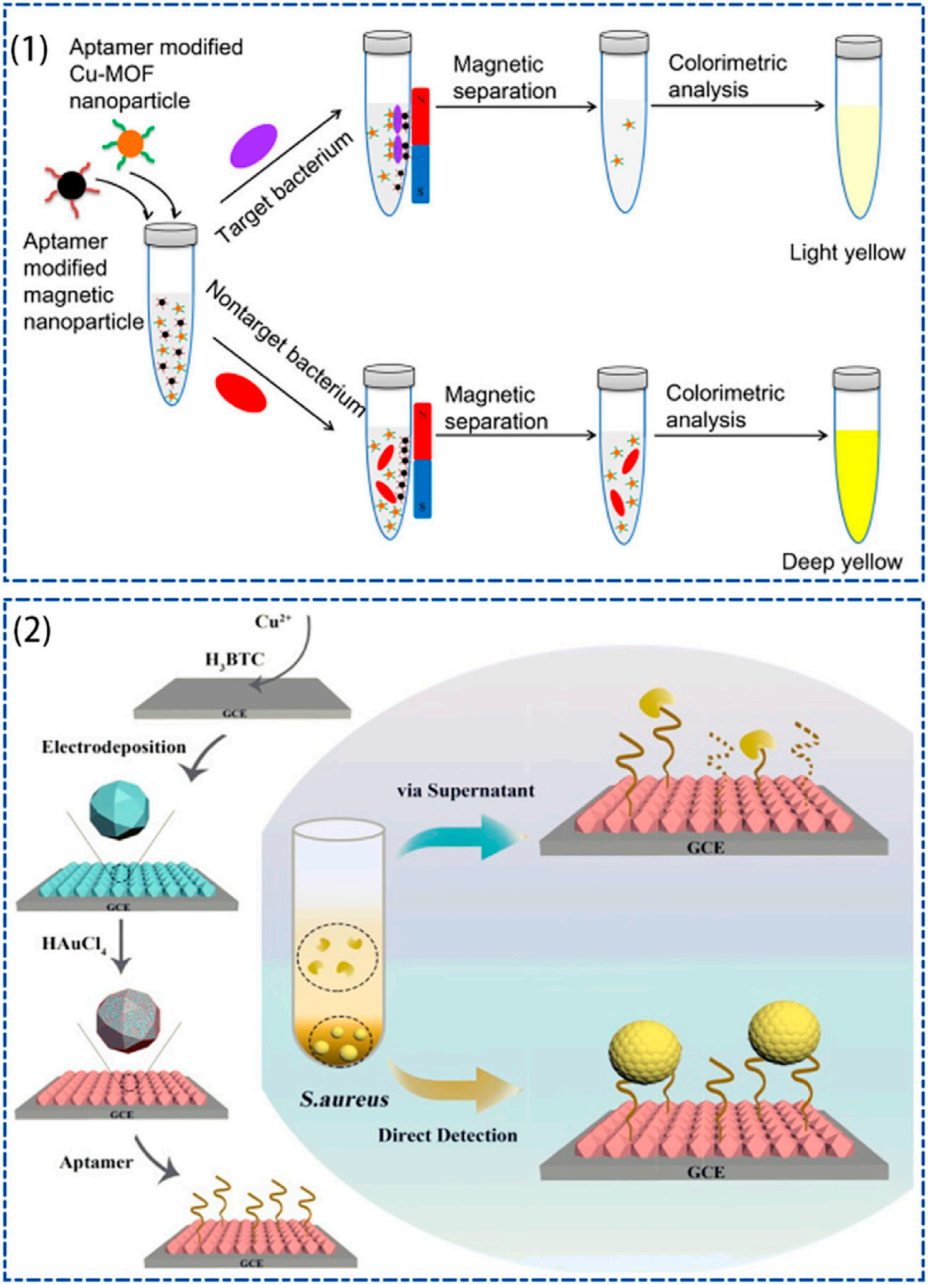
Schematic diagram of Cu-MOF-based for the detection of *S. aureus*, (1) schematic diagram of aptamer-modified Cu-MOF for colorimetric detection of *S. aureus* (Wang S et al., 2017), (2) schematic diagram of DNA/AuNPs/Cu-MOFs electrochemical sensing platform preparation and *S. aureus* detection (Sun et al., 2021).

Wang S et al. (2017) developed a simple, sensitive, and selective colorimetric assay for *S. aureus* based on Cu-MOF. Cu-MOF nanoparticles have been shown to possess peroxidase-like activity and can catalyze the yellow color development reaction of 3.3′,5.5′-tetramethylbenzidine (TMB) in the presence of H_2_O_2_. Furthermore, the presence of abundant amine groups on the surface of Cu-MOF nanoparticles allows for a simple modification of the *S. aureus* aptamer, therefore ensuring the selective recognition of *S. aureus* by Cu-MOF nanoparticles. By combining the Cu-MOF-catalyzed chromogenic reaction with aptamer recognition and magnetic separation, the detection limit for *S. aureus* detection was 20 CFU/mL. Recently, Sun et al. (2021) also designed an electrochemical biosensor for *S. aureus* detection using Cu-MOF, which reduced the detection limit of *S. aureus* to 5.2 CFU/mL (Figure 5).

The potential value of Cu-based MOFs for the adsorption and detection of food contaminants has yet to be fully explored. From the data we have collected (Table 3), there are relatively few studies on Cu-based MOFs as adsorbent materials for food contamination. Cu-based MOFs are mostly electrochemically sensed when used as sensors, probably due to the redox properties of Cu ions being used primarily for electrochemical sensing (Table 4). Furthermore, according to a related report (Liang et al., 2022), the antibacterial properties of Cu ions should not be overlooked and need to be further developed and utilized in food packaging and additives.

**TABLE 3:** Cu-based MOFs as adsorption materials for food contamination.

Cu-based MOFs	Target analytes	Type of food contaminants	Application	Sample	LOD/AC	Ref
**Chemical contamination**						
DMP-Cu	Hg(II)	Heavy metals	SPE	rice	249.5 mg/g	Song et al. (2021)
Cu_3_(BTC)_2_	Pb(II)	Heavy metals	MSPE	Rice, pig liver, Tea leaves	0.29 μg/L	Wang et al. (2015)
[Hf_6_(*μ*_3_OH)_8_(OH)_8_][(Cu_4_I_4_) (ina)_4_]_2_·22DMF	I	Food Quality	MSPE	-	329 mg/g	Hu et al. (2022)
**Biological contamination**						
Cu-BTC MOF-derived porous	aflatoxin B1	Mycotoxins	-	vegetable oils	16.6667 mg/g	Ma et al. (2021)
NH_2_-Cu(MOF)	Endotoxin	Bacterial Contamination	-	water	2500Eu/mg	Rasuli et al. (2021)

LOD, limit of detection; AC, adsorption capacity.

**TABLE 4:** Cu-based MOFs as sensors for food contamination detection.

Cu-based MOFs	Target analytes	Type of food contaminants	Application	Sample	LOD/AC	Ref.
**Chemical contamination**						
Cu-TCPP	Pb(II)	Heavy metals	Electrochemical sensor	river water	1.7 nM	Ma P et al. (2022)
PtNPs@Cu-MOF	Pb(II)	Heavy metals	Electrochemical sensor	tap water and orange juice	0.2 pmol/L	Dong et al. (2022)
Cu-MOFs	Hg(II)	Heavy metals	Electrochemical sensor	nilk	4.8 fM	Zhang X X et al. (2020)
Cu-TCPP	chloramphenicol	Pesticide/Veterinary Residues	FRET sensor	milk	0.3 pg/mL	Yang et al. (2018)
Cu-MOF derivative/SPE	Levofloxacin	Pesticide/Veterinary Residues	Electrochemical sensor	urine	0.17 μM	Zhou et al. (2022)
CDs@Cu-MOFs	thiophanate-methyl	Pesticide/Veterinary Residues	Fluorescent sensor	apple, pear, and tomato	3.67 nmol/L	Yu et al. (2022)
AuNPs/Cu-N-C	nitrite	Additives and Dyes	Electrochemical sensor	sausage	0.01 μM	Liu et al. (2022)
**Biological contamination**						
Cu-TCPP MOF	ochratoxin A (OTA)	Mycotoxins	Electrochemical sensor	apple juice and orange juice	0.08 fg/mL	Qiao et al. (2021)
MIP/Au@Cu-MOF/N-GQDs/GCE	patulin	Mycotoxins	Electrochemical sensor	apple juices	0.0007 ng/mL	Hatamluyi et al. (2020)
HKUST-1	ochratoxin A	Mycotoxins	Fluorescent sensor	corn	2.57 ng/mL	Hu et al. (2017)
Cu-MOF/Fe_3_O_4_-GO	zearalenone (ZEN)	Mycotoxins	Electrochemical sensor	cereal, maize powder and rice flour	23.14 ng/mL	Zeng et al. (2022)
CuMOF	aflatoxin B1	Mycotoxins	Electrochemical sensor	wheat flour	8.3 × 10^−4^ ng/mL	Jahangiri et al. (2022)
N-Cu-MOF	deoxynivalenol (DON)	Mycotoxins	Electrochemical sensor	wheat	0.008 ng/mL	Wen et al. (2021)
DNA/AuNPs/Cu-MOFs	*Staphylococcus aureus*	Bacterial Contamination	Electrochemical sensor	urine	5.2 CFU/mL	Sun et al. (2021)
Cu-MOF	*Staphylococcus aureus*	Bacterial Contamination	Colorimetric sensor	milk	20 CFU/mL	Wang S et al. (2017)
Ab/Cu_3_(BTC)_2_-PANI/ITO	*Escherichia coli*	Bacterial Contamination	Electrochemical sensor	water	2 CFU/mL	Gupta et al. (2019)
Cu-MOF NP	*Escherichia coli*	Bacterial Contamination	Colorimetric sensor	milk	2 CFU/mL	Duan et al. (2020)

LOD, limit of detection; AC, adsorption capacity.

### 3.3 Application of Zr-based MOFs in food contamination adsorption and detection

Zr-based MOFs generally have better mechanical stability because they have high connectivity of Zr group clusters and strong Zr-O coordination bonds in the framework (Kong and Li, 2021). Zr-based MOFs mainly refer to carboxylic acid Zr-based MOFs, which exhibit better water stability than other reported MOFs. The main feature of Zr-MOFs is the high valence state of the Zr(IV) cation, which gives a strong affinity between Zr(IV) and carboxylic acid O (Xia et al., 2021). UiO-66 was first synthesized by Cavka et al. (2008), and this MOF consists of a Zr^4+^ node provided by Zr_6_O_4_(OH)_4_ and a 1,4-benzenedicarboxylic acid ligand. Its thermal, mechanical and hydrolytic stability all up to 450°C is mainly attributed to the stable coordination bond between Zr^4+^ and the carboxylate ligand.

#### 3.3.1 Zr-based MOFs as adsorption materials for food contamination

##### 3.3.1.1 Adsorption of chemical contamination by Zr-based MOFs

**FIGURE 6:**
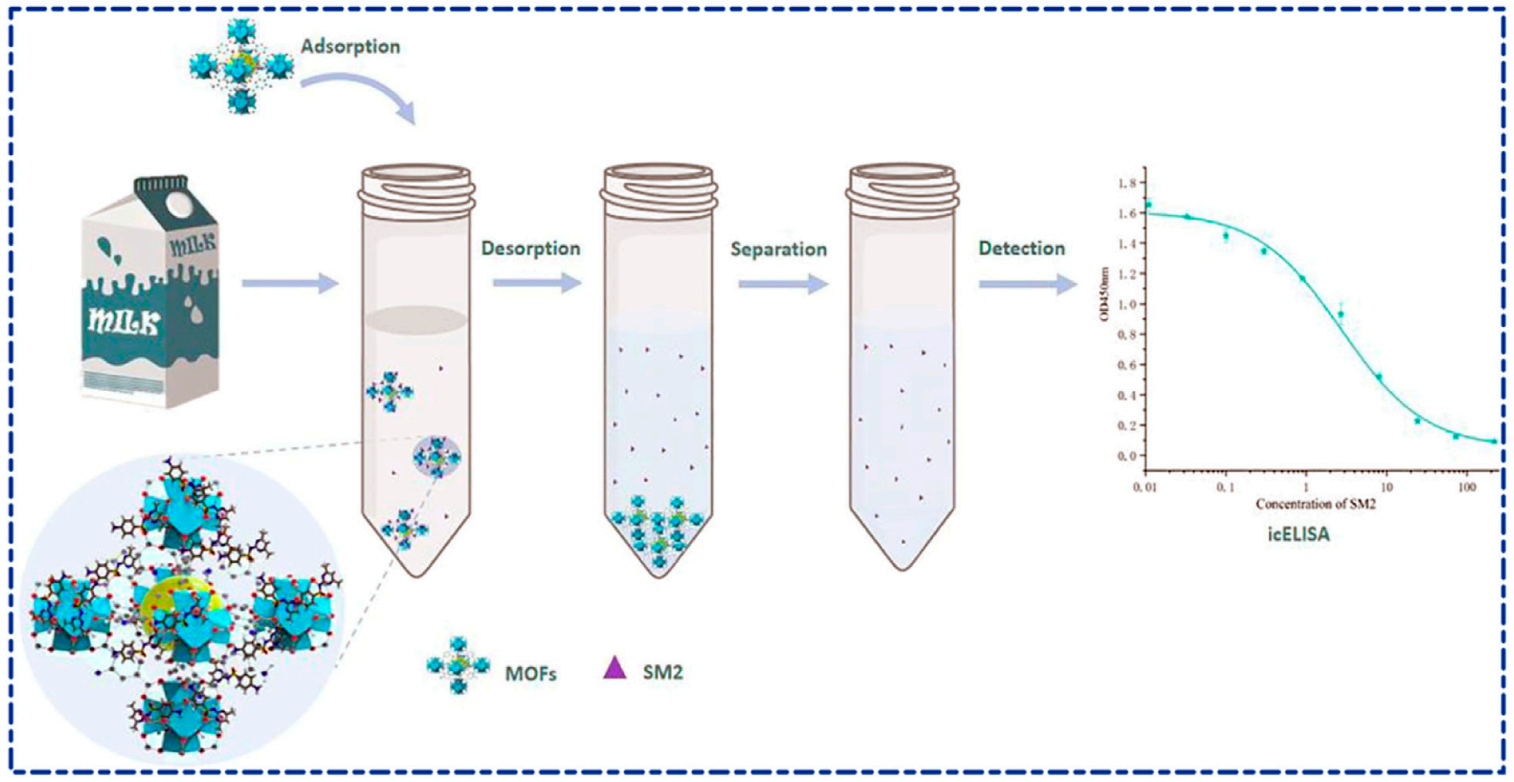
Schematic diagram of ELISA detection of SM_2_ adsorbed by MOFs in milk (Wang et al., 2022).

Zr-MOFs have high chemical, thermal and mechanical stability relative to other materials and are widely used in practical applications. The stability of Zr-MOFs can be attributed to the oxygenophilic nature of the Zr_6_ clusters and Coulombic gravitational force between these Zr cationic clusters and the anionic ligands (Ahn et al., 2016). UiO-66 as Zr-based MOF has been widely investigated by researchers for its excellent properties, high chemical and thermal stability, and diverse modification modifications (e.g., -NH_2_ (Tang et al., 2021), -NO_2_ (Wang et al., 2020), -OH (Sun et al., 2020)). Wang et al. (2022) developed a MOF-ELISA assay for a rapid detection of sulfadimethoxine (SM_2_) in milk (Figure 6). The saturation adsorption of SM_2_ by UiO-66-NH_2_ was found to be 139.64 mg/g at 298K, which was much higher than that of the other two MOFs materials, and reached 178.80 mg/g when the temperature was increased to 318 K. A close correlation was observed between MOF-ELISA and LC-MS/MS (*R* ^2^>0.99). Adsorption kinetic study revealed that the adsorption kinetic model of MOFs was more consistent with pseudo-second-order adsorption kinetics, indicating that the rate-limiting step of the adsorption process was chemisorption (Chen et al., 2020). There were two patterns for MOFs to adsorb SM_2_. The adsorption of SM_2_ in MOFs could be affected by both the internal diffusion of the crystal particles and the interaction of the material boundary layer with SM_2_ (Wu et al., 2019). The slope of the pre-sorption curve larger than that of the mid-and late-sorption curve indicated that MOFs had sufficient adsorption sites in the early stage of adsorption. When the adsorption entered the second stage, the active sites of MOFs were gradually occupied, and the adsorption rate started to decrease (the slope of the adsorption curve was smaller than that of the first stage). When the adsorption equilibrium was reached, the adsorption capacity no longer varied with the adsorption time, indicating that the internal pore structure had a significant effect on the adsorption of SM_2_.

**FIGURE 7:**
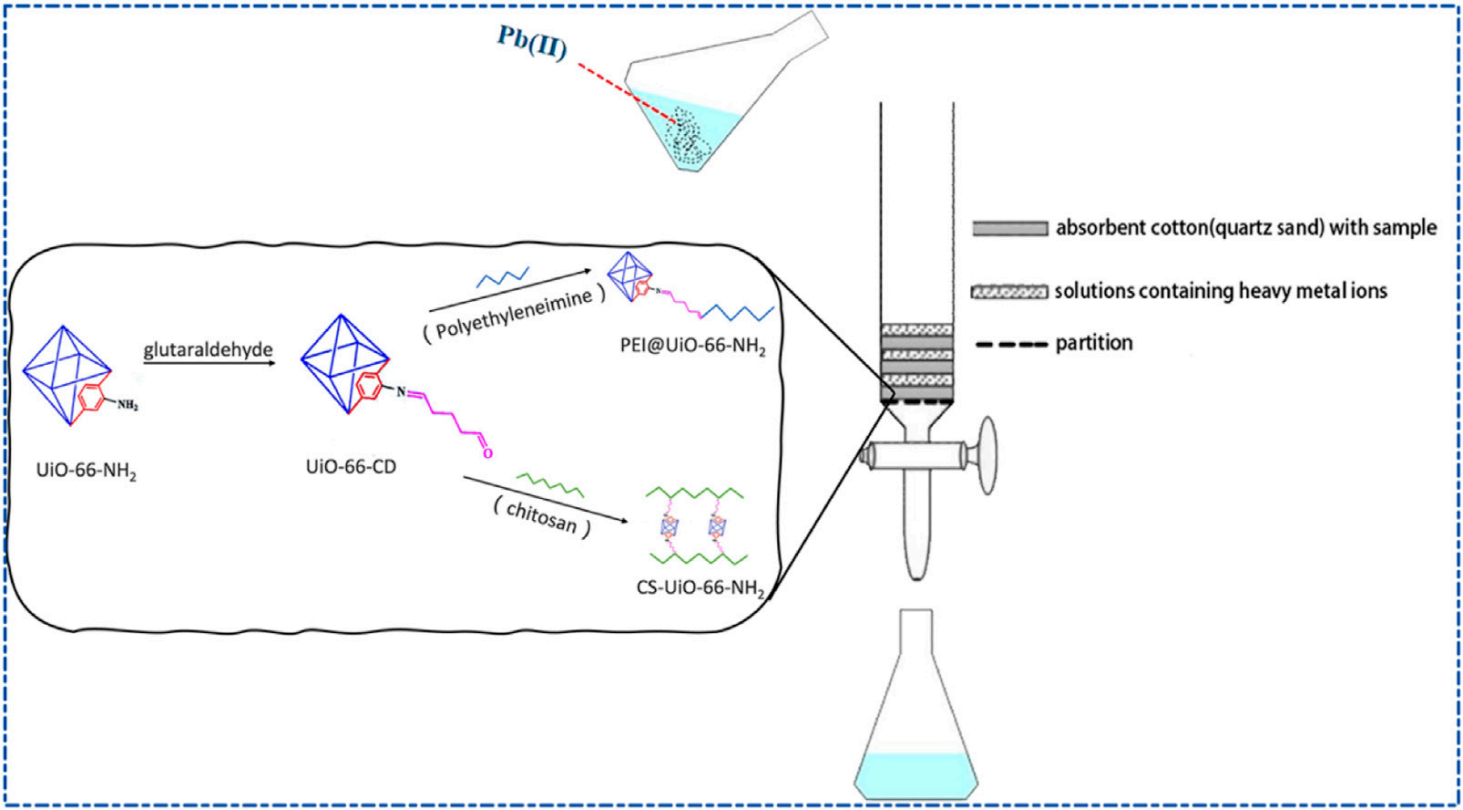
Schematic diagram of the preparation process and removal of Pb(II) from wastewater for CS-UiO-66-NH_2_ and PEI@UiO-66-NH_2._

In addition to the detection of veterinary drug residues in food, Zr-based MOFs also have a great adsorption ability for heavy metal contaminants. Hu et al. (2022a) proposed a composite MOFs material (CS-UiO-66-NH_2_) using chitosan (CS) modified UiO-66-NH_2_, which solved the problem of agglomeration of UiO-66-NH_2_ in practical applications and increased its contact area with heavy metal ions, achieving a stronger adsorption capacity. The UiO-66-NH_2_ attached to the main chain of CS by covalent bonding did not reduce the active adsorption sites but noticeably improved the dispersibility and preserved the morphology of the MOFs as much as possible. The adsorption performance of Pb(II) showed that the maximum adsorption capacity reached 555.56 mg/g. After five cycles, the adsorption capacity still reached 74.8% of the first adsorption capacity, indicating that the composite MOFs material had an excellent reusability. In another study, Hu et al. (2022b) modified UiO-66-NH_2_ with polyethyleneimine (PEI), and the prepared composite PEI@UiO-66-NH_2_ exhibited even better adsorption performance for Pb(II) (Figure 7).

##### 3.3.1.2 Adsorption of biological contamination by Zr-based MOFs

Most conventional MOFs are difficult to be biologically active as a chemical material. Recently, Wei et al. (2022) used the graded porous properties of MOFs to synthesize PCN-222 with peroxidase-like activity, which derives its biological activity from Fe-TCPP (TCPP = tetrakis (4-carboxyphenyl) porphyrin), and the peroxidase-like activity can be further enhanced by loading Pd nanoparticles onto PCN-222. The composite material was used for the adsorption and removal of AFB1 from edible oil, with an adsorption and removal rate of 96.52% by testing. Deoxynivalenol (DON) is difficult to enter the pore interior of MOFs because of large spatial site resistance of molecules. Cheng et al. (2021) designed and developed a stable adsorbent material BUT-16 based on cage-like Zr(IV)-MOFs with high performance, and found that DON molecules could interact with Zr_6_ clusters in BUT-16 to form a large number of hydrogen bonds. However, BUT-17, which has the same spatial configuration, has a tiny difference between the ligands used to build MOFs (ligand H4CPTTA for building BUT-17 has a benzene ring at its center, and ligand H4BCPIA for building BUT-16 has a pyridine ring at its center). As a result, the O atom in the adsorbed DON molecule can only form a hydrogen bond with the H atom on the ligand in BUT-17, therefore showing a tremendous difference in the adsorption. Meanwhile, such a slight difference in the ligands made the angle of the planar rings in the ligands different and allowed the DON molecules to enter the Zr_6_ cluster in BUT-16 more easily. By testing in water samples, BUT-16 demonstrated a high adsorption capacity of 46 mg/g for DON molecules.

#### 3.3.2 Zr-based MOFs as sensors for food contamination detection

##### 3.3.2.1 Detection of chemical contamination by Zr-based MOFs

Based on molecular imprinting technique, Amiripour et al. (2021) prepared a fluorescent sensor (MIP/Zr-MOF) consisting of molecularly imprinted polymers (MIPs) wrapped with Zr-based MOFs for detecting chloramphenicol (CAP) in milk and honey. Zr-LMOFs have specific binding sites for CAP recognition and therefore a high selectivity and sensitivity to CPA when encapsulated by MIPs. For the detection of CAP in real milk and honey samples, the linear range was 0.16–161.56 μg/L, and the LOD was 0.013 μg/L. Meanwhile, the recoveries measured by this method were in the range of 96%–105%, which generally in accordance with the recoveries by LC-MS/MS (95%–105%). This indicated that the functionalized LMOFs can be well applied to the analysis of CAP residues in food safety assessment. In order to detect organophosphorus pesticide residues, Gao et al. (2021) designed a novel non-enzymatic electrochemical sensor with terephthalic acid as the ligand and Zr as the metal ion in combination with electroreduced graphene oxide (rGO). The Zr-OH group in the Zr-BDC-rGO composite showed a high affinity for phosphate groups, enabling it to selectively recognize methyl parathion (MP) and have a specific adsorption capacity. The sensor was tested to have a wide linear range from 0.001–3.0 μg/mL, and the detection limit for MP was as low as 0.5 ng/mL.

##### 3.3.2.2 Detection of Zr-based MOFs against biogenic contamination

Li Z et al. (2019) developed a novel water-stable luminescent metal-organic framework material, Zr-LMOFs (Zr-CAU-24). Due to the intense blue fluorescence of Zr-CAU-24, the sensitive and rapid detection of AFB1 in water without antibodies or biomolecular modifications was achieved. Using this porous nanomaterial, the detection limit of AFB1 in walnut and almond beverages was as low as 19.97 ppb (64 nM) with a high recovery (91%–108%). The structural stability of the Zr_6_ ion cluster in water plays a crucial role in mycotoxin detection. Li Z et al. (2021) recently reported a composite sponge material based on Zr-LMOFs, which was loaded on a melamine (MF) sponge using *in situ* hydrothermal synthesis. Such a bifunctional hybrid sponge not only retained the great mechanical processing properties of MF sponge, but also had a high tolerance to large deformation. At the same time, the pore structure of the preserved Zr-LMOFs crystals allowed the guest molecules to enter well inside the cavity, enabling the detection of AFB1 with a relatively high sensitivity (LOD = 1.6 ppb) and a wide linear range of 0.1–50 μM. It was further found that the analyte-sensor interaction played a crucial role in the efficient quenching. Moreover, AFB1 was rapidly enriched in the cavity of Zr-LMOF, producing stronger analyte-sensor interaction and higher-order π-π interaction, which could accelerate the electron transport and generate a greater quenching effect for sensitive detection. Therefore, based on this property, it is feasible to develop hybrid materials based on Zr-LMOF for rapid detection and removal of toxic and hazardous substances.

The Zr_6_ clusters of Zr-based MOFs give them high chemical, thermal and mechanical stability compared to other materials. As can be seen from Tables 5, 6, research for chemical and biological contamination applications is prevalent. High sensitivity and a wide linear detection range are achieved by compounding with different functional materials. At the same time, continuously optimized materials have improved the adsorption capacity for contaminants. In particular, the use of bioactive substances to modify MOFs makes them well suited for the adsorption and detection of biological contamination.

**TABLE 5:** Zr-based MOFs as adsorption materials for food contamination.

Zr-based MOFs	Target analytes	Type of food contaminants	Application	Sample	LOD/AC	Ref.
**Chemical contamination**						
CS-UiO-66-NH_2_	Pb(II)	Heavy metals	-	water	555.56 mg/g	Hu et al. (2022a)
PEI@UiO-66-NH_2_	Pb(II)	Heavy metals	-	wastewater	692.80 mg/g	Hu et al. (2022b)
MOF-545	Pb(II)	Heavy metals	SPE	chickpea, bean, wheat, cherry juice	1.78 μg/L	Tokalıoğlu et al. (2017)
SS-NH_2_-UiO-66–5	Pb(II)	Heavy metals	-	wastewater	186.14 mg/g	Li et al. (2022)
UiO-66-IT	Hg(II)	Heavy metals	-	water	580 mg/g	Awad et al. (2021)
MOF-808	I	Food Quality	-	-	2.18 g/g	Chen et al. (2020)
UiO-66	phytohormones	Pesticide/Veterinary Residues	SPE	vegetable samples	0.01–0.02 ng/mL	Yan et al. (2018)
Cotton@UiO-66	phenoxy herbicides	Pesticide/Veterinary Residues	SPE	Soil, cucumber, tap water	0.1–0.3 mg/L	Su et al. (2020)
UiO-66-NH_2_	sulfamethazine (SM_2_)	Pesticide/Veterinary Residues	ELISA	milk	178.80 mg/g	Wang et al. (2022)
Am-UiO-66	nitrosamines	Additives and Dyes	SPME	artificial saliva solution	2.61–6.12 ng/L	Huang et al. (2018)
**Biological contamination**						
PCN-222	aflatoxin B1	Mycotoxins	-	oil	-	Wei et al. (2022)
UiO-66-NH_2_ derived porous (HMONs)	aflatoxin (AFT)	Mycotoxins	SPE	corn, soybean, millet, rice	82.3 mg/g	Yang L et al. (2022)
BUT-16	deoxynivalenol	Mycotoxins	-	water	46 mg/g	Cheng et al. (2021)
UiO-66-NH_2_@MIP	aflatoxins	Mycotoxins	SPE	grain	2.7 μg/kg	Liang et al. (2020)

LOD: limit of detection; AC, adsorption capacity.

**TABLE 6:** Zr-based MOFs as sensors for food contamination detection.

Zr-based MOFs	Target analytes	Type of food contaminants	Application	Sample	LOD/AC	Ref.
**Chemical contamination**						
MIP/Zr-MOF	chloramphenicol (CAP)	Pesticide/Veterinary Residues	fluorescent sensor	milk, honey	0.013 μg/L	Amiripour et al. (2021)
Zr-BDC-rGO	methyl parathion (MP)	Pesticide/Veterinary Residues	Electrochemical sensor	vegetable	0.5 ng/mL	Gao et al. (2021)
BUT-12 and BUT-13	antibiotics	Pesticide/Veterinary Residues	Fluorescent sensor	water	10 ppb	Wang et al. (2016)
Zr-LMOF	parathion-methyl	Pesticide/Veterinary Residues	Fluorescent sensor	water	0.438 nM	He et al. (2019)
Eu/Zr-MOF	tetracycline	Pesticide/Veterinary Residues	Fluorescent sensor	water	0.00092 μg/mL	Li Y et al. (2021)
Zr−TPPS MOFs	Cu(II)	Heavy metals	colorimetric sensor	water	4.96 nM	Hou et al. (2022)
UiO-66-NH_2_	Cd(II)	Heavy metals	Electrochemical sensor	water	0.3 μg/L	Wang Y et al. (2017)
Dye@UiO-66-OH/PVA MMM	trimethylamine	Food Quality	colorimetric sensor	chicken	80 ppm	Ma P et al. (2022)
Eu/Zr-based MOF	acrylamide	Additives and Dyes	Fluorescent sensor	fried potatoes, cookie crumbs	24 nM	Yao et al. (2021)
**Biological contamination**						
Zr-CAU-24	aflatoxin B1	Mycotoxins	Fluorescent sensor	walnut and almond beverages	19.97 ppb (64 nM)	Li X et al. (2019)
Zr-LMOF/MF	aflatoxin B1	Mycotoxins	Fluorescent sensor	water	1.6 ppb (0.058 μM)	Li Z et al. (2021)
UiO-66-NH_2_	aflatoxin B1	Mycotoxins	Fluorescent sensor	corn, rice, milk	0.35 ng/mL	Jia et al. (2020)
UiO-67/GR	*Salmonella typhimurium*	Bacterial Contamination	Electrochemical sensor	milk	5 CFU/mL	Dai and Ye (2019)

LOD, limit of detection; AC, adsorption capacity.

### 3.4 Application of other MOF materials in food contamination adsorption and detection

In addition to the several metal-based MOFs mentioned above, many other MOFs materials have been investigated for the adsorption and detection of food contamination. Among them, Co-based MOFs are fast developing and have been gradually applied to food detection. Zhang X et al. (2022) found that Co-based MOFs exhibited good stability under alkaline conditions and could be utilized as bio-inspired nanoenzymes, which in turn led to the successful development of a portable detector that could be used to detect acetamiprid residues. Liu et al. (2019) developed an ultra-sensitive and highly selective Co-based MOF and nitrogen-rich COFs for the detection of the antibiotic ampicillin (AMP) using the electrochemical sensing platform Co-MOF@COFs, which has great potential for food safety applications. Fu et al. (2022) developed an Al-based MOF probe that uses the fluorescence signal and ratio of the scattered intensity signal for efficient and sensitive detection of ciprofloxacin (CIP). Since NH_2_-MIL-53 is a nanoscale particle, it has both fluorescence and second-order scattering signals. The intrinsic fluorescence signal of CIP overlaps with NH_2_-MIL-53, which enhances the background signal and is not conducive to detection. Therefore, the authors modified it with 8-hydroxy-2-quinolinecarboxaldehyde (HQCA), and the modified HQCA-MIL-53 showed almost no fluorescence properties compared to NH_2_-MIL-53, but still exhibited scattering properties. Ghiasi et al. (2022) prepared an aptamer functionalized material based on magnetic CoFe_2_O_4_@SiO_2_@MIL-101(Cr)-NH_2_ for magnetic solid phase adsorption (MSPE) of pesticide residue acetamiprid from fruit juice. The adsorbent utilized the synergistic effects of the high specific surface area of MIL-101(Cr), magnetic properties of CoFe_2_O_4,_ and specific molecular recognition of the aptamer, and showed a strong performance in the extraction of aminopyralid with a maximum adsorption capacity of 3.5 mg/g. In recent years, cyclodextrin metal-organic frameworks (CD-MOFs) based on cyclodextrin (CD) have been widely investigated not only for their typical edible, regenerative, and biodegradable properties, but also for their large surface area, controllable pore size, and high adsorption properties on gases. Majd et al. (2022) successfully prepared a cross-linked polypropionitrile/γ-cyclodextrin-metal-organic framework (PAN/γ-CD-MOF) electrospun nanofibers based on CD-MOFs, which were used as an efficient adsorbent for extracting multiple herbicides from cereal samples prior to HPLC-UV analysis. This method was effectively applied for trace analysis of herbicides in wheat, rice, and barley samples. In addition, CD-MOFs were also used for sensing assays. Recently, Chen Y et al. (2022) proposed a novel sensor for determinating luteolin (3′, 4′, 5, 7-tetrahydroxyflavonoids, LU) by preparing a glassy carbon electrode (MoO_3_-PEDOT/CD-MOF/GCE) using γ-CD-MOF, MoO_3_ and a flexible conducting polymer poly (3,4-ethylenedioxythiophene) (PEDOT). The *in situ* growth of PEDOT on MoO_3_ nanorods effectively improves the stability, dispersion, and electrical conduction efficiency of PEDOT. Meanwhile, the cavities in CD-MOFs can absorb a large amount of LU and increase the local concentration of LU on the electrode surface, which further amplifies the current signal. Under the optimal conditions, the sensor exhibits a wide linear range of 0.4 nM–1800 nM and a low detection limit of 0.1 nM (S/N = 3).

## 4 Conclusion

In summary, MOFs have shown great potential for the adsorption and detection of food contaminants. This paper highlights recent insightful research results on the adsorption and detection of food contaminants by Zn-based, Cu-based and Zr-based MOFs, describes how they work and discusses their application in the adsorption and sensing detection of chemical and biological contaminants in terms of stability, adsorption capacity and sensitivity. Despite the many advances that have been made, there are still many challenges in their practical application. Firstly, although MOFs materials can be synthesized with different pore sizes depending on the length of the organic ligand and the size of the metal ionophore, most MOFs studied so far are more suitable for the adsorption of small molecules (e.g. heavy metals and other chemical contaminants) and have bulk rejection of large molecules, so precise control of pore size and volume for specific applications (including adsorption performance and sensing selectivity) is still to be development. Secondly, the functional versatility, structural flexibility and large specific surface area of MOF materials are their greatest advantages as adsorbent materials, compared to commercially available materials such as zeolites and activated carbon. However, some MOF materials still lack stability in aqueous solutions, which is an urgent issue in the development and utilization of MOFs. The stability of MOFs can be improved by compounding with different stabilizing materials. Furthermore, an important limitation of MOFs is the use of non-renewable raw materials for their synthesis, but this problem has recently been solved by the manufacture and use of γ-cyclodextrin-based MOFs. Currently, γ-cyclodextrin-MOFs are being developed as a new green and edible MOF and will be considered for future research in food packaging and food additives. Finally, some of the already commercialized MOFs (e.g. ZIF-8, UiO-66, HKUST-1) can be developed for specific applications depending on the metal ion. Zn-based and Zr-based MOFs materials have been widely employed in the detection of chemical contamination because of their stable skeleton structure as well as in fluorescence detection and analysis. Due to the redox reaction of Cu^2+^/Cu, Cu-based MOFs are electrochemically active, and the large number of electroactive sites in Cu-MOFs facilitates signal amplification, so they are used more in electrochemical sensors. Meanwhile, Cu ions have been studied more in food preservation due to their broad-spectrum antibacterial activity, such as loading mesoporous HKUST-1 on chitosan quaternary ammonium salt films to achieve good ethylene adsorption and antibacterial capacity (Nian et al., 2022). It can be seen that MOFs are a promising research material that can play a valuable role in food contamination adsorption and detection through targeted modifications and functionalization, thus enhancing food safety for rapid and accurate risk assessment.
